# Spatial Variation in General Medical Services Income in Dublin General Practitioners

**DOI:** 10.1155/2011/971231

**Published:** 2011-06-15

**Authors:** Conor Teljeur, Alan Kelly, Tom O'Dowd

**Affiliations:** Department of Public Health & Primary Care, Trinity College, Dublin 2, Ireland

## Abstract

The general medical services (GMS) scheme provides care free at the point of use for the 30% most economically deprived section of the population and the elderly. Almost all people of over-70-year olds are eligible for the GMS scheme potentially directing resources away from those most in need. The aim of this study is to analyse the relationship between practice GMS income and deprivation amongst Dublin-based general practitioners (GPs). The practice GMS income in Dublin was analysed in relation to practice characteristics including the number of GPs, catchment area population, proportion of over-70-year olds in the catchment area, catchment deprivation, number of GMS GPs within 2 km, and average GMS practice income within 2 km. Practice GMS income was highest in deprived areas but is also a valuable source of income in the least deprived areas. The capitation rate for over-70-year olds provides an incentive for GPs to locate in affluent areas and potentially directs resources away from those in greater need.

## 1. Introduction

General practitioners (GPs) can be paid by a variety of methods: capitation fees, fee for service, and salary being the most common [1]. Fee for service tends to result in overtreatment of patients, whereas capitation tends to encourage undertreatment and preventive measures [2]. In addition to the method of payment, financial incentives have been used in various countries to attract GPs to locate in disadvantaged areas [3]. The incentives are typically in the form of payments calculated based on the socioeconomic characteristics of practice catchments. However, targeting on the basis of areas may result in a failure to reach many deprived individuals as not all deprived people live in deprived areas and not all people in a deprived area are necessarily deprived [4].

In Ireland, and unlike the United Kingdom, GPs are paid through a combination of fee for service for private patients and capitation fees for eligible patients funded by the state. The capitation rates are linked to the age and sex of the patient and the distance they live from the GP practice. Approximately thirty percent of the Irish population is covered for GP care under the general medical services (GMS) scheme, while the remaining 70% of the population pay full fees to access GPs [5]. Although covering only a third of the population nationally, the GMS scheme accounts for 57% of GP income and is highly valued by GPs as it is superannuated and attracts staffing subsidies. Nearly all general practices in Ireland now combine GMS and private practice. 

Patient eligibility for the GMS scheme is determined on a means-tested basis, and GPs are reimbursed on a capitation basis for treating GMS patients. Prior to the 2002 general election, the government decided that GMS eligibility should be extended to all over-70-year olds. Having announced their intentions prior to entering contract talks, the government found itself in a weak negotiating position. Only the fees for the newly eligible over-70-year olds were under negotiation, and the existing coverage and capitation fees were maintained. A much higher capitation rate was agreed for treating the newly eligible over-70-year olds. Universal eligibility for over-70-year olds was removed at the end of 2008 although it was estimated that 95% of over-70-year olds would continue to be eligible for free care [6]. The cessation of universal cover for over-70-year olds was accompanied by a new unified capitation fee for all over-70-year olds to end the differential fee [7].

While in situ, the differential capitation rate for the over-70-year olds may have worked to attract GMS GPs to affluent areas with a large elderly population, but a previous study by the authors established that variation in access to GP services by deprivation in urban parts of Ireland is relatively small [8]. It appeared that the GMS scheme had not unfairly distorted the distribution of GPs in relation to deprivation. However, the provision of free care to over-70-year olds largely irrespective of income may be directing resources away from those most in need. The aim of this paper is to analyse the relationship between GP income derived from the GMS scheme and deprivation amongst Dublin-based GPs, taking into account the population distribution of over-70-year olds.

## 2. Methods

### 2.1. Setting

County Dublin is a predominantly urban area with a population of 1.2 million as of the 2006 census, equivalent to 28% of the national population. Seven percent of the Dublin population is over 70 years of age, slightly below the national figure of 7.7%. There are 322 electoral divisions (EDs) in county Dublin with a mean population of 1244 (range 76–32288). Thirty percent of the Dublin EDs are in the most deprived decile nationally and 22% in the least deprived decile nationally [9]. Half of the over-70-year olds in Dublin are split equally between least deprived decile and the most deprived decile of EDs.

Since 1989, under the general medical services (GMS) scheme, the state has contracted GPs to provide care free at the point of use for the poorest 30% of the population on a capitation basis. Coverage of the GMS scheme has varied between 28.1% and 32.5% of the population from 1999 to 2008. Since 2005, coverage has been increasing gradually. At the time of the study, an estimated 95% of over-70-year olds were eligible for the GMS scheme. Approximately 96% of GP practices nationally provide care under the GMS scheme although within Dublin the figure is closer to 85%. From 2001 to 2008, the capitation fee for a patient over-70-year old without prior eligibility for the GMS was €672 compared to €147 and €162 for previously eligible (i.e., based on means testing) males and females over-70-year olds, respectively. Since January 2009, there has been an average capitation fee of €290 for all over-70-year olds eligible for the GMS scheme. The gross weekly income limits for GMS eligibility are €184, €201.50, and €700 for a single person aged under 66, 66 to 69 and over 70 years, respectively. Allowances for dependent children apply to those under 70 years of age.

### 2.2. Data

The primary care reimbursement service (PCRS) publishes the list of GPs in receipt of GMS payments [10]. The most recent year available was 2009 with data distinguishing between practice support and GMS fees. Multiple GPs in the same practice may be in receipt of practice support which is used to subsidise the cost of a practice manager and nursing and secretarial staff. The addresses of GPs were obtained from a variety of sources including the Irish College General Practitioners (ICGP), CervicalCheck, and the Irish Medical Directory [11–13]. GP addresses were then geocoded to a point location.

EDs were assigned deprivation scores based on the 2006 national deprivation index [9]. The deprivation index is similar in structure to that of the Townsend deprivation index used extensively in the UK. The index combines four indicators of material deprivation into a score: unemployment, low social class, car ownership, and local authority housing. The deprivation score can also be expressed in deciles to label the most deprived 10% of EDs. For this study, the deprivation score is used in preference to the deciles to capture the variability that can occur within a single deprivation decile. As the deprivation score is positively skewed, the most deprived decile spans a wide range of scores.

The characteristics of practice catchments were estimated using data from local EDs. The ED practice was used as the centre of the practice catchment. The deprivation and population contribution of each ED to a catchment profile reduced with increasing distance from the practice ED. The distance weighting was estimated using a distance decay function developed in a previous study [8]. A practice-specific deprivation score was computed using the nearest 20 EDs to the practice ED. The deprivation scores were weighted using a combination of the weights generated by the distance decay function multiplied by the ED populations. Practice population of over-70-year olds was computed based on distance weighting alone. Catchment area population is not a measure of practice list size but acts as a proxy for demand in a practice catchment area.

Patients with GMS eligibility can choose which GMS GP they attend within their locality. Restrictions apply as there is a cap on how many GMS patients a GP can have in his list at any one time, but there is a scope for choice of practice on the part of the patient. A large number of GMS GPs in a locality can represent oversupply and reduce incomes. As a proxy for competition, we determined the number of GMS GPs and the average GMS income of GPs within 2 km of each practice.

### 2.3. Statistical Analysis 

To acknowledge the clustered data structure and the large number of clusters (*n* = 202) involved, total practice income was analysed using a Bayesian hierarchical model with practice (level 1) nested within electoral division (level 2). Total practice income was transformed to the log_e_ scale to address both skewness and heteroscedasticity in the distribution of income and then back transformed for purposes of reporting results. The Bayesian modelling was carried out via JAGS [14] using the *R* statistical program [15] with the *rjags* package (v 2.2.0-3) employing 3 chains. The number of MCMC iterations was 400,000 with a burn-in of 20,000. All model coefficients had successfully converged based on a Gelman & Rubin statistic of 1.04 and the Heidelberger & Welch test offered by *Coda *[16]. Predictions of total practice income were simulated using the distribution of the dependent variable conditional on the estimated parameters from the model. As is customary in Bayesian model reporting, to assess the significance of the model coefficients, a 95% Bayesian credible interval is reported in lieu of a *P* value. The *mgcv* package (v 1.7-2) within *R* was used with the default choice of smoothing spline to visualise the dependency between predicted total practice income and deprivation.

## 3. Results

Five hundred and eighty-four Dublin-based GPs are listed as having some GMS income in 2009. Address records could not be found for three GPs leaving 581 included in this analysis. After address coding, a total of 383 distinct practices were identified which were distributed across 202 EDs. The average number of GMS patients per GMS GP in Dublin is 528 compared to a figure of approximately 540 nationally. The overall mean practice income was €271,572 which comprised practice support (mean = €49,738) and GMS fees (mean = €221,847). The mean estimated percentage of patients over 70 years old per practice was 7.6% (range 1.5%–14.5%). Sixty eight percent of practices had only one GP in receipt of GMS fees with only 6 practices having 5 or more GMS GPs. The median number of GPs within 2 km of a practice was 21. The median and interquartile ranges (IQR) for the main practice level and ED level characteristics are given in Table 1.

Using a regression model, deprivation and proportion population over-70-year olds were found not to be significant predictors of the number of GMS GPs in a practice. Thus, there was no tendency for larger practices in more or less deprived areas or in areas with high proportions of over-70-year olds.

**Figure 1:**
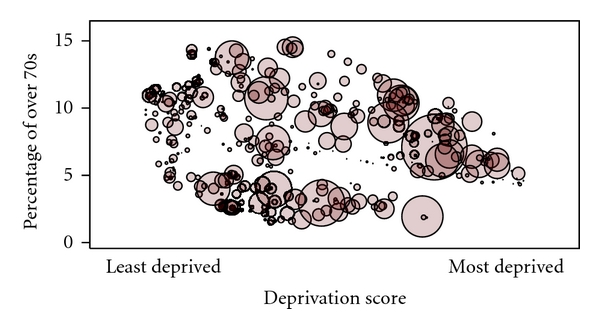
Proportion catchment population over 70 by deprivation score (circle size proportional to total practice GMS income) in county Dublin.

There is a modest trend for a decreasing proportion of over-70-year olds in the catchment population with increasing practice deprivation (see Figure 1). The size of the circles in Figure 1 is proportional to the total practice income. Few of the practices at the least deprived end of the spectrum have large GMS-derived incomes. It is likely that almost all GMS income for these practices comes from treating eligible patients of over-70-year olds. Conversely, for practices in the most deprived areas, the income comes almost entirely from GMS patients under 70 years of age.

**Figure 2:**
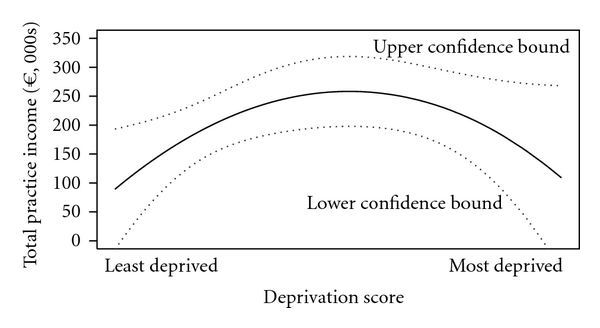
Predicted total practice GMS income by deprivation score in county Dublin.

The contribution of different predictors to total practice GMS income was estimated using a hierarchical Bayesian model (see Table 2). Deprivation is a significant predictor of practice income; however, as the relationship is quadratic, total income tends to fall again at high levels of deprivation (see Figure 2). The percentage of over 70s has, on average, no impact on total income. The number of GMS GPs within 2 km negatively impacts on total income; as anticipated, competition acts to reduce practice income.

GMS incomes in practices in the least deprived areas are lower with the largest incomes in areas with moderate levels of deprivation (Figure 2). However, low incomes are also observed in the practices with the most deprived catchments. These practices are characterised by mostly being single handed with low proportion of patients of over-70-year olds.

## 4. Discussion

GPs in Ireland are funded through a mixture of fee for service for private patients and capitation fee for publicly funded patients. Almost all over-70-year olds are eligible for state-funded care. Practices in more deprived areas have higher GMS incomes than those in affluent areas suggesting that state-funded care is both profitable and generally reaching those who can least afford to pay for care. The profitability in deprived areas stems primarily from the high volume of patients under 70 years of age.

The biggest impact on total practice GMS income is predictably the number of GPs in the practice. A larger workforce enables a bigger volume of patients to be treated. The percentage of over-70-year olds has a modest positive impact on income, and we have shown that less deprived areas have higher percentages of over-70-year olds. The level of competition from neighbouring practices has a significant negative impact on practice GMS income highlighting the contribution of supply and demand to practice income. Practice deprivation score plays a relatively minor role differentiating Ireland from UK, where population level deprivation has traditionally made an important contribution due to the explicit use of deprivation scores in primary care resource allocation.

This study has only investigated income from the GMS which represents 57% of total GP income. For practices in more deprived areas, the GMS income may well represent most if not all of practice income. In more affluent areas, on the other hand, where a significant proportion of patients pay for services, the GMS income will only represent a small portion of the total practice income. A patient over 70 is, in monetary terms, equivalent to 3 to 4 patients aged 16 to 44 years and 2 patients aged 45 to 69 years. Clearly, a small number of patients of over-70-year olds can provide a useful source of income in affluent catchment areas.

Incomes in practices serving the most deprived communities are relatively low compared to other deprived areas. It is probable that GPs working in the most deprived areas work fewer sessions in an effort to control workload and stress [17]. We were not able to adjust the results for the number of sessions or list size. Data are not available on non-GMS practice income which would provide useful counterbalance and enable an estimate of the value of GMS patients of over-70-year olds in affluent areas.

Although GP utilisation increases with age, the disparity in both capitation fee and eligibility criteria between those under and over 70 years of age is introducing inequity into the health care system. In a fixed-budget health system with limited resources, if care cannot be provided for all, then it must surely be directed to those most in need. But, by providing free GP care to those of over-70-year olds largely irrespective of the level of personal wealth, the state directs resources away from those most in need and provides a valuable income to GPs located in the least deprived areas. A small nondeprived but elderly catchment can be as valuable as a larger deprived catchment with a younger population. The combination of higher workloads and stress associated with practice in a deprived catchment and the differential capitation payments may act to encourage GPs to locate in less deprived areas. We have shown that despite the near universal cover for over-70-year olds, GMS incomes are highest in deprived areas although they are lower in the most deprived areas. It is encouraging to observe that GPs continue to locate in deprived areas. However, in the absence of universal free GP care, resources should not be channelled away from those who cannot afford to pay towards those who can.

## Figures and Tables

**Table 1:** Characteristics of Dublin GMS GP practices.

Characteristic	Median	(IQR)
Number of GPs in practice	1	(1-2)
Total practice support income (€,000s)	31.5	(3.7–74.2)
Total practice GMS fees (€,000s)	171.2	(73.9–298.1)
GPs within 2 km	21	(13–32)
Average GMS income of GPs within 2 km (€,000s)	168.5	(135.5–214.4)
Population in catchment area	3200	(2846–3961)
Over 70s in catchment area	270	(212–308)
Over 70s in catchment area (%)	8.5	(5.3–10.5)
Deprivation score	0.60	(−0.59–2.87)

**Table 2:** Model coefficients and 95% Bayesian credible intervals for predictors of total practice income in 383 practices across 202 electoral divisions.

Predictor	Coefficient	(95% credible interval)
Intercept	93,901	(773–9,065,622)
Population	0.98	(0.56–1.73)
Deprivation score	1.30	(1.05–1.62)
Deprivation score squared	0.95	(0.90–1.00)
Percentage of over 70s in practice catchment	1.04	(0.99–1.08)
Number of GMS GPs in practice	1.80	(1.56–2.05)
Number of GMS GPs within 2 km	0.82	(0.97–0.99)
